# Alkali Metal Ion Insertion in Polypyrrole Polyoxometalates for Multifunctional Actuator–Sensor–Energy Storage Devices

**DOI:** 10.3390/polym17030262

**Published:** 2025-01-21

**Authors:** Rudolf Kiefer, Ngoc Tuan Nguyen, Quoc Bao Le

**Affiliations:** 1 Conducting Polymers in Composites and Applications Research Group, Faculty of Applied Sciences, Ton Duc Thang University, Ho Chi Minh City 700000, Vietnam; lequocbao@tdtu.edu.vn; 2 Faculty of Applied Sciences, Ton Duc Thang University, Ho Chi Minh City 700000, Vietnam; nguyenngoctuan@tdtu.edu.vn

**Keywords:** PPyDBS-PT, alkali metal cations, actuator, sensor, energy storage, multifunctionality

## Abstract

Modern research technology’s goal is to produce multifunctional materials that require low energy. In this work, we have applied polypyrrole (PPy) doped with dodecyl benzenesulfonate (DBS-) with the addition of polyoxometalates (POM) such as phosphotungstic acid (PTA) forming PPyDBS-PT composites. Two different PTA concentrations (4 mM and 8 mM) were used to form PPyDBS-PT4 and PPyDBS-PT8. The higher concentration of PTA created a highly dense and compact film which can be observed from scanning electron microscopy (SEM cross-section image), and also contains fewer phosphotungstate anions (PT^3−^) inclusion (via energy-dispersive X-ray spectroscopy, EDX). Three different aqueous electrolytes, LiCl (lithium chloride), NaCl (sodium chloride), and KCl (potassium chloride), were applied to investigate how those alkali metal ions perform as typical cation-driven actuators. Cyclic voltammetry with linear actuation revealed the tendency LiCl > NaCl > KCl in view of better strain, charge density, electronic conductivity, and Young’s modulus of PPyDBS-PT4 outperformed PPyDBS-PT8. Chronopotentiometric measurements showed high specific capacitance for PPyDBS-PT4 at 260.6 ± 21 F g^−1^ with capacity retention after 5000 cycles of 88.5%. The sensor calibration of PPyDBS-PT4 revealed that the alkali cations (Li^+^, Na^+^, and K^+^) can be differentiated from each other. The PPyDBS-PT4 has multifunctional applications such as actuators, sensors, and energy storage.

## 1. Introduction

Linear conducting polymer actuators such as PPy doped with DBS^−^ are well-known PPyDBS cation-driven actuators in aqueous electrolytes with expansion at a reduction. The DBS^−^ anions are incorporated in PPy during electropolymerization at oxidation which balance the positive charges, and at reductions, the left DBS^−^ macro anions, named “immobile” due to their size, are compensated by incorporated cations with solvent through osmotic pressure [1]. Several applications are shown using PPyDBS either as a bilayer with use in micro-robotics [2], biomedical applications [3], smart textiles [4], anion- and cation-driven free-standing yarns [5], and in linear actuators with strain-up to 12% as well [6]. PPyDBS with polyethylene oxide (PEO) composite as linear actuators has also been shown for cation selective sensors and energy storage capability [7], underlining their multifunctional applications. Previous research with increasing PTA in PPyDBS polymerization showed that in an aqueous solvent, the best linear strain was obtained for PTA at 10 mM (PPyDBS-PT10), while the best specific capacitance (energy storage) was found at 5 mM PTA [8] (PPyDBS-PT5). The inclusion of PTA in conducting polymers showed from previous research [9] using PPy nano-pipes as having 1.5 to 2 times higher specific capacitance (6.2–6.8 F cm^−3^) compared to pristine PPy nano-pipes. The inclusion of PTA in poly-3,4 ethylenedioxythiophene (PEDOT) forming with PPy/phosphomolybdic acid (PMA) as an asymmetric supercapacitor revealed excellent specific capacitance of 31 F g^−1^ in comparison to PPy/PMA symmetric capacitor [10]. The addition of polyoxometalates on carbon nanotubes (polyvinyl acetate used as a binder) has also been demonstrated to enhance the specific capacitance with values at the range of 285 F g^−1^ (0.2 A g^−1^) [11]. The use of polyoxometalate addition in polymer composites for supercapacitor materials has been shown in various research [12,13].

Previous research using pristine PPyDBS revealed that Li^+^ and Na^+^ in aqueous solvents are not so different in the sensor equation and cannot be differentiated [7]. Therefore, with the addition of PTA in the electropolymerization of PPyDBS, our primary goal in this work is to study alkali cations such as Li^+^ (lithium cation), Na^+^ (sodium cation), and K^+^ (potassium cation) of three electrolytes (LiCl, NaCl, and KCl) to evaluate if those can be sensed from each other in an aqueous solvent. PPyDBS-PT composite used as an actuator, sensor, and energy storage device has not been shown before, opening the path of multifunctional applications.

The specific capacitance was also determined to evaluate the energy storage capacitance of the two samples. The difference from the previous research was to change the polymerization condition to a lower temperature (−40 °C) with PPyDBS films, as shown in recent research, leading to more dense and compact films, influencing surface conductivity and overall linear actuation properties [14]. Different PTA concentrations of previous research, such as 4 mM and 8 mM forming novel PPyDBS-PT4 and PPyDBS-PT8, are investigated.

Simultaneously, actuation and sensing have been explored in PPy either by sensing temperature [15], trailed weight [16], or electrolyte concentration [17]. The sensor separation of alkali cations such as Li^+^, Na^+^, and K^+^ using carbon nitride nanotubes [18] revealed through cation adsorption energy the tendency Li^+^ > Na^+^ > K^+^ with Li+ and Na^+^ adsorption energy being quite similar, making it difficult to separate those two cations, which is also shown for metal–polymer composites [19] and PPyDBS in recent research [20]. Using PPyDBS as a sensor from previous research shown for Li^+^, Na^+^, and K^+^, the solvation number in an aqueous solvent was found in a similar range for Li^+^ and Na^+^ ions of 4.5 to 5.5 [21], leading to similar expansion at reduction either for Li^+^ or Na^+^ cations [7]. Including PT3^−^ in PPyDBS will enhance the stored negative charges and influence the pseudo capacitance and the overall strain response, as shown before [22]. Also, varied PTA concentration in PPyDBS affects their pseudocapacitance [8]. Therefore, the addition of PT^3−^ in PPyDBS influences strain and charge density, which we assume will help in sensing different-sized cations (here Li^+^, Na^+^, and K^+^). The envisaged applications for such PPy composites are smart textiles with multifunctional materials in actuation, sensing, and energy storage needed for future use in healthcare technology. The main condition that such different PPyDBS-PT films can be compared needs a certain order that in the applied potential range (0.65 V to −0.6 V), the charging/discharging is in balance [23] following the electrochemically stimulated conformational relaxation (ESCR) model [24]. Electrochemical measurements such as cyclic voltammetry and chronopotentiometry with parallel electro-chemo-mechanical deformation (ECMD) measurements were performed. SEM microscopy, Fourier Transform Infrared (FTIR) spectroscopy, and EDX spectroscopy were performed to characterize PPyDBS-PT composites.

## 2. Materials and Methods

### 2.1. Chemicals

Pyrrole (Py, 98%) was purchased from Sigma Aldrich (Taufkirchen, Germany), distilled, and stored in the dark under nitrogen at −20 °C. Electrolytes such as lithium chloride (LiCl, 99.9%), sodium chloride (NaCl, 98%), potassium chloride (KCl > 99%), sodium dodecylbenzenesulfonate (NaDBS, technical grade), and phosphotungstic acid hydrate (PTA, PW_12_O_4_O_3_^−^, reagent grade) were supplied by Sigma-Aldrich and used as supplied. Solvents such as deionized water (MilliQ+), ethylene glycol (EG, 98%), and ethanol (technical grade) were obtained from Merck (Ho Chi Minh City, Vietnam).

### 2.2. Formation of PPyDBS with PTA

The galvanostatic electropolymerization (0.1 mA cm^−2^, 40.000 s, −40 °C) took place in a two-electrode set-up using potentiostat/galvanostat (PARSTAT 2273, Princeton applied research, Oak Ridge, TN, USA) with a cryostat (Julabo, Thermostat Bath FP40-HE, Seelbach, Germany) to control the low temperature at electropolymerization. The electrode cell contained stainless steel sheets as working electrodes (18 cm^2^) placed in the middle with the opposite of two stainless steel mesh (counter electrode). The monomer solution consisted of 0.1 M pyrrole (Py) and 0.1 M NaDBS in Milli-Q+/EG (50/50 wt.%) to obtain PPyDBS (used for FTIR measurements). Adding 4 mM PTA in the monomer solution led to PPyDBS-PT4, and 8 mM PTA formed PPyDBS-PT8 films. After electropolymerization, the PPyDBS-PT composite films were removed from the stainless steel working electrode. The films were afterward washed in Milli-Q+ to remove excess PTA and NaDBS. Additional washing with ethanol was applied to remove unreactive Py. The procedure was repeated four times, and then the films were dried in the oven at 40 °C (2 mbar) for 24h. The film thickness of wet (24h storage in aqueous electrolytes) PPyDBS-PT4 was found at the range of 24.5 ± 1.8 µm and PPyDBS-PT8 had 19.8 ± 1.1 µm. The thickness of the wet samples was measured by an electronic micrometer gauge meter (SAGARTEX, STT001, 0.001 mm sensitivity, Dhaka, Bangladesh).

### 2.3. Linear Actuation Measurements

The PPy films were cut in the length of 1.1 cm and 0.1 cm width with PPyDBS-PT4 in the wet state weighing 250.3 ± 19 µg, and PPyDBS-PT8 found reduced to 217.1 ± 17 µg. The two film samples were put under load (~1 g, 9.8 mN) with one side fixed on a static arm that contained gold contacts (5 mm in length), while the other end was fixed (clamp size 5 mm) on the force sensor (TRI202PAD, Panlab, Barcelona, Spain) that was connected to the movable stage (smallest steps are 0.5 µm). The linear muscle analyzer was a homemade device [25] that measures mass change. To obtain the length change expressed in strain ε (Δl/l × 100 (%)), those were determined through the calibration of how much mass/length changes (stiffness factor k was calculated to the elastic modulus of mass/µm aka stress/strain) were obtained before measurements and after measurement. The k factor served as a calibration tool for calculating the mass change in length. The length of the films between the two clamps was set to 1 mm. The films (PPyDBS-PT4 and PPyDBS-PT8) were connected as the working electrode to the potentiostat (Biologic PG581, Seyssinet-Pariset, France) with a counter electrode, a platinum sheet (6 cm^2^) and an Ag/AgCl (3M KCl) reference electrode. Before measurements, the films were stretched at 1% strain for 12 h in the aqueous electrolytes (0.2 M: LiCl, NaCl, and KCl). Cyclic voltammetry (scan rate 5 mV s^−1^) was conducted in real time. The signals from the potentiostat and the length change were written in an in-home software [25]. 

Chronopotentiometric measurements of the PPyDBS-PT4 film samples were conducted using varied current densities of ±0.2 A g^−1^, ±0.4 A g^−1^, ±0.8 A g^−1^, ±2 A g^−1^, ±4 A g^−1^, and ±8 A g^−1^ with a same charge density of ±40 C g^−1^. The PPyDBS-PT8 current densities varied from ±0.23 a g^−1^, ±0.46 A g^−1^, ±0.92 A g^−1^, ±2.3 A g^−1^, ±4.6 A g^−1^, and ±9.2 A g^−1^ with a same charge density of ±46 C g^−1^. From each PPyDBS-PT4 and PPyDBS-PT8 film, at least three independent films were polymerized and investigated, with the results presented in mean values and standard deviations. The specific capacitance *C_s_* of the composite films is obtained through Equation (1) [26].

(1) $$C_{s}=\frac{i}{-slope\cdot m}$$

From the potential time curves (ΔV/Δt) at each chronopotentiogram, the slope (obtained from linear fit) of the discharge cures (after IR drop) at each applied current density (*i*/*m*) was obtained. Previous research used the determination of the specific capacitance from cyclic voltammetry and chronopotentiometry, revealing similar values for the PEDOT-based films [27]. Another measurement obtained from the chronopotentiogram (potential *E* against time *t*) is the electrical energy Ue by integrating the discharged curves at each applied current density *i*/*m*, given in Equation (2).

(2) $$U_{e}\;\left(t\right)=\frac{i}{m}\int E\left(t\right)dt$$

### 2.4. Characterizations

After film formation, the SEM surface and cross-section images of PPyDBS-PT4 and PPyDBS-PT8 were made through Vega Tescan (Tescan Orsay Holding, Brno-Kohoutovice, Czech Republic). EDX spectroscopy (Oxford Instruments with X-Max 50 mm^2^ detector, High Wycombe, PA, USA) of the surface images before and after the washing steps were performed. Additionally, after actuation cycles (~100 cycles of cyclic voltammetry), the film sample was oxidized for 3 min at 0.65 V in the applied electrolyte, and then the film was cut by washing and drying the cut piece, and from the cross-section image, EDX spectroscopy was performed. The leftover film was reduced at −0.6 V for 3 min, cut, washed, and dried, and EDX spectroscopy of the cross-section was performed. For each PPyDBS-PT4 and PPyDBS-PT8 in the three electrolytes LiCl, NaCl, and KCl, the same procedure at oxidation and reduction after actuation cycles was carried out. FTIR measurements (Bruker Alpha with platinum ATR, Billerica, MA, USA) of the PPyDBS, PPyDBS-PT4, and PPyDBS-PT8 films in oxidized form (+0.65 V) and PTA (pressed in KBr pellets) at wavelength range 2000 cm^−1^–700 cm^−1^ were accomplished. The electronic surface conductivity of the film samples (dry state) was obtained by applying the four-point probe conductivity meter (Jandle, Model RM2, Leighton Buzzard, UK).

## 3. Results and Discussions

Having PPyDBS-based conducting polymers, the addition of PTA, also known as polyoxometalate, has two purposes: to bring more negative charges (PT^3−^) in PPy beside the large DBS^−^ anions to trigger higher linear actuation. The second purpose, hence PTA, is a catalyst and light oxidant [28,29] and, in combination with PPy, also serves as an anti-oxidant [30], making those PPyDBS-PT composites more stable against over-oxidation. Our approach in this work is the use of those multifunctional PPyDBS-PT composites as actuator–sensors applying three electrolytes, LiCl, NaCl, and KCl, given the alkali cations (Li+, Na+, and K^+^) to find out if those can be differentiated from each other. Their energy storage capability was another feature to investigate as PPyPOM composites in previous research have been found to be a promising candidate [31]. 

### 3.1. Polymerization and Characterization of PPyDBS-PT4 and PPyDBS-PT8

**Figure 1:**
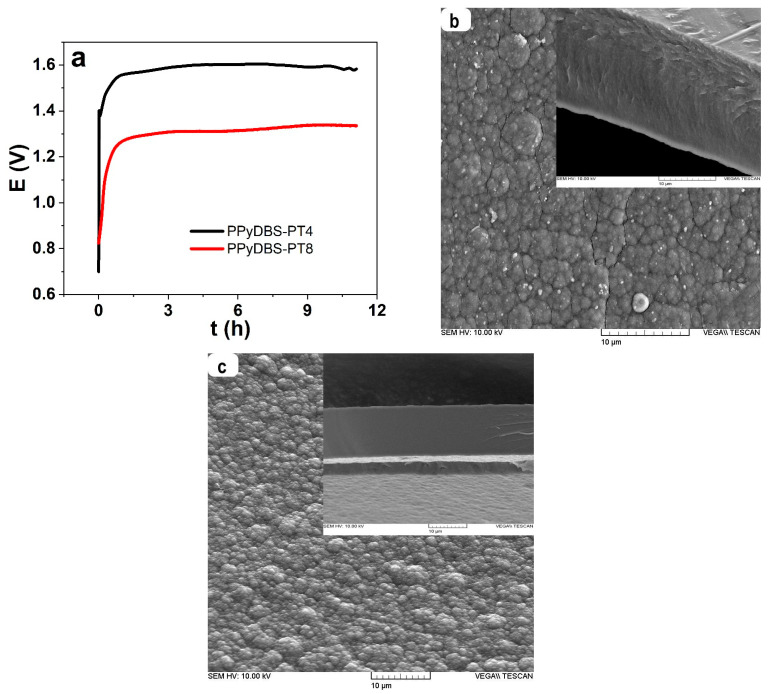
(**a**) Galvanostatic electropolymerization forming PPyDBS-PT4 (black line) and PPyDBS-PT8 (red line) in a two-electrode cell containing monomer solution and PTA (either 4 mM or 8 mM) showing potential against time t. The obtained PPyDBS-PT4 film surface with an inset cross-section image (scale bar 10 µm) are displayed in (**b**) and those from PPyDBS-PT8 in (**c**).

PTA are well-known polyanions that are generally applied to form a suspension of carbon particles, such as carbon-derived carbon or multiwall carbon nanotubes in an aqueous solution [32]. Using only pyrrole and PTA as electrolytes, the electropolymerization did not lead to any stable PPy-PT films, while the addition of NaDBS formed stable PPyDBS-PT films. The electropolymerization curves of PPyDBS-PT4 and PPyDBS-PT8 are presented in Figure 1a. The SEM surface with the inset of the cross-section image is shown for PPyDBS-PT4 in Figure 1b and those from PPyDBS-PT8 in Figure 1c.

From the polymerization curve in Figure 1a, the PPyDBS-PT4 (4 mM PTA in electropolymerization) potential increased to 1.6 V after 4.2 h and then decreased at the end of polymerization time (11.1 h) to 1.54 V. A decrease in potential at galvanostatic polymerization shows the deposition of PPyDBS-PT4 on a stainless steel working electrode. In the case of PPyDBS-PT8, the potential compared to PPyDBS-PT4 is clearly reduced at the end at polymerization curve 1.33 V. The main reason for the much lower potential of PPyDBS-PT8 is the properties of PTA serving as a catalyst [29] that affect the film growth found in previous research with increasing PTA concentration reducing film thickness [8].

**Figure 2:**
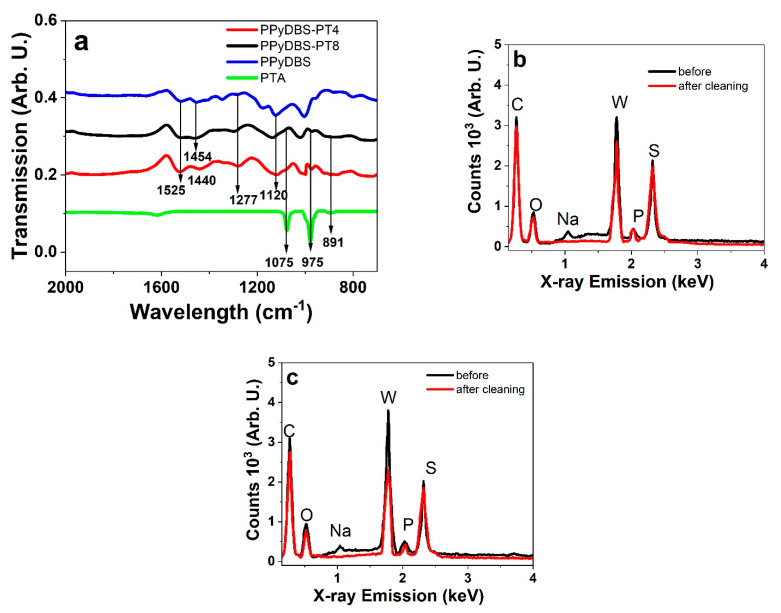
(**a**) FTIR measurements (2000 cm^−1^–700 cm^−1^) of PPyDBS-PT8 (black line), PPyDBS-PT4 (red line), PPyDBS (blue line), and PTA (green line). EDX spectroscopy at oxidized state (0.65 V) before (black line) and after cleaning (red line) of PPyDBS-PT4 shown in (**b**) and PPyDBS-PT8 in (**c**).

The SEM surface image shows the typical cauliflower formation for both PPyDBS-PT films (Figure 1b,c) [33]. The overall thickness of the cross-section image (Figure 1b, inset) of PPyDBS-PT4 showed 20.3 µm, and PPyDBS-PT8 (Figure 1c, inset) had 16.5 µm thickness. After storage in an aqueous electrolyte, the film thickness increased in the range of 23–25%, with PPyDBS-PT4 showing 24.5 ± 1.8 µm and PPyDBS-PT8 (increase in aqueous electrolyte around 15%) had 19.8 ± 1.1 µm. The film density of PPyDBS-PT4 was 0.928 ± 0.07 g cm^−3^, and PPyDBS-PT8 showed a higher density of 0.997 ± 0.09 g cm^−3^. Those differences are reflected in the denser cross-section image of PPyDBS-PT8 in Figure 1c (inset). The other difference between PPyDBS-PT4 and PPyDBS-PT8 is the electronic conductivity that was found to be nearly three times higher for PPyDBS-PT4 with 36 ± 1.9 S cm^−1^ and 11.2 ± 0.7 S cm^−1^ for PPyDBS-PT8. Further analysis, such as the FTIR and EDX spectroscopy of the PPyDBS-PT films directly after formation are presented in Figure 2a–c, respectively.

The typical PPy bands are shown in Figure 2a at 1525 cm^−1^ (N-H bending [33]) with PPyDBS, and PPyDBS-PT8 had a band at 1454 cm^−1^ shifting to 1440 cm^−1^ for PPyDBS-PT4 (PTA accelerating shifts [34]) with those bands in the range of 1500–1400 cm^−1^ belonging to C=C and C-C bending vibrations [35]. The other bands are all shown in PPy films found at 1277 cm^−1^ (C-H stretching vibration in the literature shown at 1281 cm^−1^ [36]) and 1120 cm^−1^ (C-H bending modes [37]). The PTA bands at 1075 cm^−1^ (1080 from the literature [38]) that refer to P-O stretching vibrations are also reflected in PPyDBS-PT8 and PPyDBS-PT4 as a weak shoulder. The 975 cm^−1^ band represents the W-O stretching vibration [38], and the 891 cm^−1^ band belongs to the bending vibrations of W-O-W bonds [39]. The FTIR spectrum in Figure 2a confirmed that PT^3−^ was incorporated in PPyDBS-PT8 and PPyDBS-PT4. Further analysis of element composition (EDX spectrum) of PPyDBS-PT4 and PPyDBS-PT8 directly after polymerization (before washing) and after washing are presented in Figure 2b and c, respectively.

The typical elements described in Figure 2b,c, including carbon (C) at 0.27 keV, oxygen (O) at 0.52 keV, sodium (Na) at 1.04 keV, tungsten (W) at 1.78 keV, phosphor (P) at 2.04 keV, and sulfur (S) at 2.32 keV. After cleaning, the element of Na was rinsed and disappeared within PPyDBS-PT4. After washing, the tungsten (W) signal intensity was also reduced by 20%, meaning that some on-surface weak adsorbed PT^3−^ were removed. The reduction in the intensity of the elements in Figure 2b after cleaning confirms such. A similar tendency is shown in Figure 2c of PPyDBS-PT8 directly after polymerization (before) and after cleaning the weak surface-bound elements, shown as well before [22]. The main difference in comparison to PPyDBS-PT4 is the much stronger tungsten signal before and after the cleaning of PPyDBS-PT8 (Figure 2c) with a decrease in intensity at the range of 40%, hints to other processes involved rather than removal of unbound elements. The PTA in the electropolymerization serves as a catalyst and light oxidant; the PPyDBS-PT8 film is much denser than PPyDBS-PT4. We assume that due to the more compact PPyDBS-PT8 film, even at higher PTA, a smaller amount of PT^3−^ in comparison to PPyDBS-PT4 is incorporated. It can be deduced that if more PTA is included in the monomer solution, higher catalyze effects (reduction in polymerization potential in Figure 1a) occur at the polymerization. Therefore, a denser packed PPy leads to reduced capacity of PT^3−^ (nearly 12% less PT^3−^ in the estimation of tungsten peak intensity) compared to PPyDBS-PT4. 

The PPyDBS films (length 1.1 cm, width 0.1 cm, and thickness 26 µm) weight in dry state was 166 ± 13 µg (density 0.58 g cm^−3^), PPyDBS-PT-4 had 206.6 ± 17 µg (dry state) and PPyDBS-PT8 weight in dry state was 183.8 ± 16 µg. Overall, the reduction in weight in view of PT^3−^ inclusion from weight comparison revealed an 11% reduction for PPyDBS-PT8, similar to the observation of EDX spectroscopy where 12% was estimated.

### 3.2. Linear Actuation Studies of PPyDBS-PT Samples

PPyDBS-based films are typical cation-driven actuators (expansion at a reduction) in aqueous electrolytes, in general, as shown by the dependence on applied electrolytes (size of cations and solvation numbers) in previous research [7] with strain at the range of 3–6%. Table 1 compares different hydration numbers and the radius of those cations (Li^+^, Na^+^, and K^+^).

In general, there is a tendency to larger the ions and their hydration number as shown in the following order (Table 1) of K^+^ > Na^+^ > Li^+^, and the ionic mobility is reduced. As shown for Li^+^ with only 4 H_2_O as the hydration number, those ions can move more freely in an aqueous solvent than the larger and higher hydrated ions (K^+^). From previous research [21], the hydration number inside PPyDBS was determined through electrochemical quartz crystal microbalance with the results that the hydration is kind of reversed in comparison to aqueous solvent, shown in Table 1 for N_PPy_ with Li^+^ > Na^+^ > K^+^. Our main interest lies in analyzing how the film density and the addition of PT^3−^ in PPyDBS forming PPyDBS-PT4 and PPyDBS-PT8 influence the linear actuation properties. Electrochemical techniques such as cyclic voltammetry and chronopotentiometry with linear actuation measurements were conducted. For each PPyDBS-PT sample with the three different electrolytes, at least three independent from each polymerized film were measured, with the results presented in mean values and standard deviations.

### 3.3. Cyclic Voltammetry

**Figure 3:**
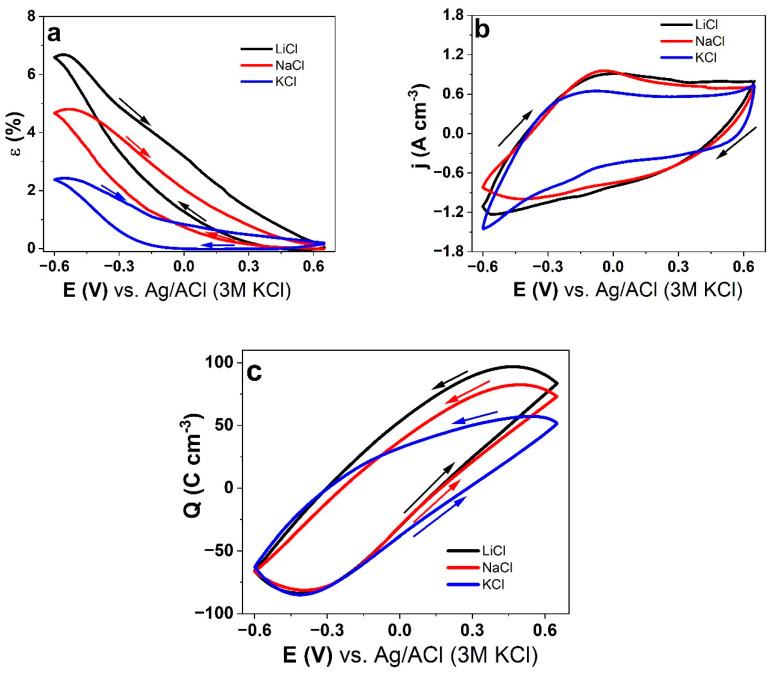
Cyclic voltammetry (scan rate 5 mV s^−1^, 3rd to 4th cycle) with linear actuation measurements in aqueous electrolytes LiCl (black line), NaCl (red line), and KCl (blue line) showing for PPyDBS-PT4 (**a**) strain curves, (**b**) current density, and (**c**) charge density against the potential E (0.65 V to −0.6 V). The arrows indicate the direction of the scan.

Cyclic voltammetry (scan rate 5 mV s^−1^) in combination with linear actuation measurements at the potential range 0.65 V to −0.6 V of PPyDBS-PT4 showing strain curves of the three electrolytes LiCl, NaCl, and KCl are shown in Figure 3a. The current density potential curves are displayed in Figure 3b, and the charge density potential loops (coulovoltammetry) are shown in Figure 3c. 

The strain curves (Figure 3a) of PPyDBS-PT4 have main expansion (strain) at a reduction (cation-driven actuator) following the tendency for the cation incorporation of the electrolytes, LiCl > NaCl > KCl. EDX measurements after cyclic voltammetry linear actuation have been made to analyze if we can observe any element changes at oxidation (0.65 V) and reduction (−0.6 V), with those results for PPyDBS-PT4 presented in Figure S1a–c. The typical element signals in Figure S1a–c, similar to Figure 2b and c having tungsten (W), are phosphor signals (P) showing that PT^3−^ in PPyDBS-PT4 are included with no change appearing at oxidation/reduction. At oxidation (0.65V) in Figure S1a using LiCl (Li too small to be detected), a small chloride (Cl) signal at 2.64 keV is shown, which was not found at reduction (−0.6 V). The tungsten, carbon (C), oxygen (O), and sulfur (S) signals (evidence that DBS^−^ incorporated in PPy) do not change much at oxidation/reduction. In the case of NaCl (Figure S1b), the small chloride signal is presented, and at a reduction, the sodium (Na) at 1.04 keV is detected with the Cl signal reduced. It reveals the typical cation-driven actuation, as shown in Figure 3a. The PPyDBS-PT4 using KCl as electrolyte with the EDX spectrum in Figure S1c has a similar tendency to the previous one, while a new signal at 3.31 keV of element potassium (K) only appears at reduction. Therefore, the PPyDBS-PT4 shown for all the applied electrolytes is purely cation-driven, with the minor chloride signal not participating in linear actuation properties. The reason for such chloride element detection at oxidation was found in previous research [42], especially dual cation and anion movement for small anions applied, which have been detected in PPyDBS. 

The current density curves of LiCl and NaCl are similar in shape, while KCl showed lower current density. The oxidation (reduction) waves of PPyDBS-PT4 at LiCl applied are found at 0.0 V (−0.15 V), NaCl at −0.04 V (−0.24 V), and KCl at −0.07 V (−0.22 V). The PPyDBS-PT4 using aqueous LiCl electrolyte showed the best linear actuation and had nearly 1.8 times better electronic surface conductivity than the KCl electrolyte. 

The coulovoltammetric response of PPyDBS-PT4 (Figure 3c) showed a close loop for all three applied electrolytes, ensuring the charging/discharging is balanced [23]. Only under this condition can linear actuation be compared according to the ESCR model [24]. Table 2 lists the strain and charge densities of PPyDBS-PT4 when applying the LiCl, NaCl, and KCl electrolytes in an aqueous solvent. The presented results in Table 2 are the mean values with a standard deviation of at least three samples of the PPyDBS-PT4 films.

Either strain, charge density, or electronic conductivity follows a similar trend of the applied electrolytes of LiCl > NaCl > KCl of the cation-driven PPyDBS-PT4 linear actuators (Table 2). The charge density of PPyDBS-PT4 applying KCl was 1.3 times lower than LiCl, and the electronic conductivity was found to be 1.8 times lower if KCl was applied. The elastic modulus determined after actuation (calculated from stiffness factor k) revealed for LiCl and NaCl nearly 3.4 times reduction from before to after actuation. In the case of KCl, the elastic modulus (Table 2) reduced slightly at the range of 1.2 times. The reduction in elastic modulus directly impacts strain, as shown by previous research [43].

**Figure 4:**
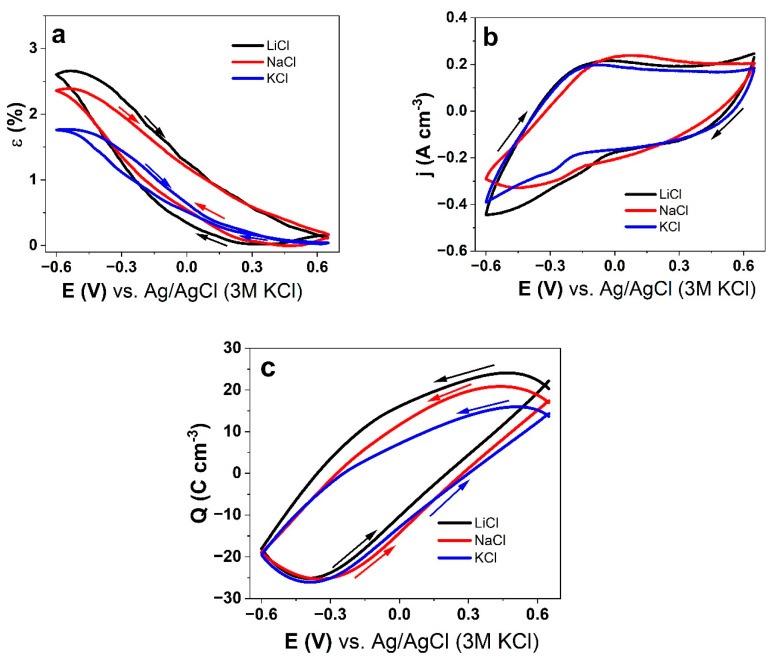
Cyclic voltammetry (scan rate 5 mV s^−1^) combined with linear strain measurements of PPyDBS-PT8 using three different aqueous electrolytes LiCl, NaCl, and KCl at the potential range (E) of 0.65 V to −0.6 V. The strain ε is shown in (**a**), the current density in (**b**), and the charge density in (**c**). The arrows in the figures show the direction of the scan.

The PPyDBS-PT8 films underwent the same cyclic voltammetric measurements applying the same aqueous electrolytes (LiCl, NaCl, and KCl) with the strain shown in Figure 4a, current density curves in Figure 4b, and charge density in Figure 4c against the potential E (0.65 V to −0.6 V).

Similarly to Figure 3a of PPyDBS-PT4, PPyDBS-PT8 in Figure 4a shows a similar tendency for all the applied electrolytes’ main expansion at a reduction with LiCl > NaCl > KCl. The strains of LiCl and NaCl, as shown in Figure 4a, are in a similar range, and the strain differences are not as striking as seen in Figure 3a. The EDX spectrum of the cross-section images is made from the PPyDBS-PT8 films after actuation cycles (~100 measurements) with the films oxidized (+0.65V) and reduced (−0.6 V), with the results for LiCl, NaCl, and KCl presented in Figure S2a–c. The elements carbon (C: 0.27 keV), oxygen (O: 0.52 keV), tungsten (W: 1.78 keV), phosphor (P: 2.04 keV), and sulfur (S: 2.32 keV) are identical with those observed in Figure 2c. As described before, the tungsten signal stays at oxidation and reduction in similar intensities, revealing that no PT^3−^ are expulsed during the redox reaction [44]. The Li element, due to its size, is not detected in the EDX spectrum, but for NaCl, clear evidence of the Na signal is observed at reduction (−0.6 V) in Figure S2b and KCl with potassium (K: 3.31 keV) in Figure S2c. Similarly to PPyDBS-PT4, small amounts of chloride (Cl: 2.64 keV) are observed at oxidation, but there is no evidence that anion incorporation leads to expansion at oxidation.

The current density (CV shapes) of PPyDBS-PT8 (Figure 4b) has 3.5 times lower values than PPyDBS-PT4 (Figure 3b). Minor oxidation (reduction) waves are observed for LiCl at −0.03 V (−0.18 V), for NaCl at 0.01 V (−0.28 V), and for KCl at −0.1 V (−0.29 V). The differences in the PPyDBS-PT4 current densities (Figure 3b) are only slightly different in the position of the oxidation/reduction waves. The overall charge density potential curves in Figure 4c show close loops of the PPyDBS-PT8 films for all the applied aqueous electrolytes, verifying that charging/discharging is in control. Table 3 compares the results of strain, charge density, electronic conductivity, and elastic modulus (before and after actuation cycles).

When comparing the strain of PPyDBS-PT8 (Table 3) with PPyDBS-PT4 (Table 2) for LiCl, those are 2.5 times reduced, as for NaCl, nearly 2 times reduced and in the case of KCl only 1.3 times lower. The main reason might be that the much stiffer PPyDBS-PT8 films with elastic modulus two times higher for all the applied electrolytes directly influence the strain. PPyDBS-PT films belong to faradaic actuators [45], and the charge density at reduction given by PT^3−^ and DBS^−^ promotes a higher amount of counter-ions here in solvated cation entrance for PPyDBS-PT4 with nearly 12% more PT^3−^ in comparison to PPyDBS-PT8. Additionally, the lower electronic conductivity after the actuation of the PPyDBS-PT films in aqueous electrolytes has been documented before for PPyDBS-related films [7]. It was also found that with increasing PTA concentration in PPyDBS polymerization, the surface conductivity does not increase but decreases [8], which is why PTA as a catalyst and light oxidant affects the surface morphology of the PPyDBS-PT films.

In summary, the PPyDBS-PT4 films have much better strain, charge density, conductivity, and lower elastic modulus results than PPyDBS-PT8. Further investigations into their ability to store energy are presented in the next section.

### 3.4. Energy Storage of PPyDBS-PT4 and PPyDBS-PT8

**Figure 5:**
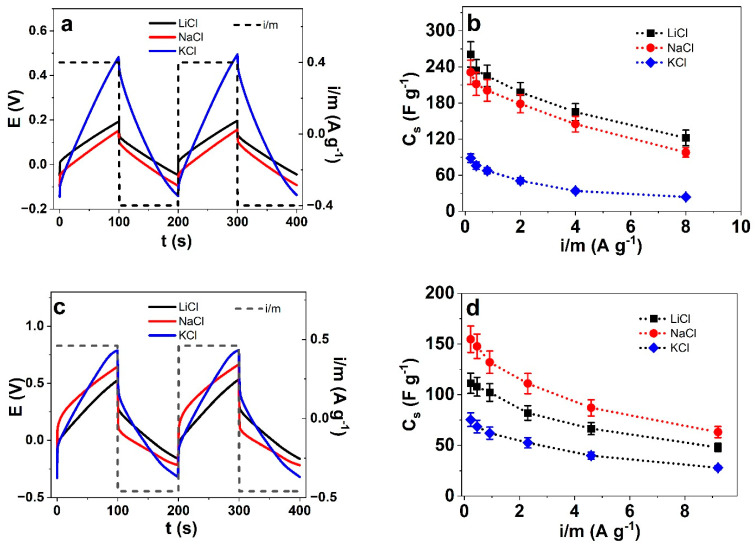
Chronopotentiometric measurements in aqueous electrolytes LiCl (black line, ■), NaCl (red line, ●), and KCl (blue line, ◆) presenting PPyDBS-PT4 within (**a**) potential E (against Ag/AgCl (3M KCl)) time t curves (3rd and 4th cycles) at ±4.0 A g^−1^ (dashed black line) and in (**b**) the specific capacitance C_s_ calculated from Equation (1) against current densities i/m (±0.2 A g^−1^–±8.0 A g^−1^). The PPyDBS-PT8 films of the potential time curves are presented in (**c**) and the specific capacitance against the current densities i/m (±0.23 A g^−1^–±9.2 A g^−1^) are displayed in (**d**).

Pseudocapacitors such as polypyrrole are well known to store higher charge densities [46] than electrical double-layer capacitors, such as carbon nanotubes. The addition of PTA or other polyoxometalates (POM) in PPy has been shown before [29] and is designed for energy storage materials. A combination of different POMs and PPys obtained through chemical oxidation, such as phosphomolybdate PMo_2_@PPy, showed specific capacitance at 294.1 F g^−1^, and phosphotungstate PW_12_@PPy reached a specific capacitance of 341 F g^−1^ in H_2_SO_4_ [9]. It was also found that in the combination of PPy through electropolymerization with the addition of PTA, the ionic conductivity increased with salt concentration compared to PPy without PTA [47]. Chronopotentiometric measurements of PPyDBS-PT4 (current densities ± 0.2 A g^−1^ to ±8 A g^−1^ (constant charge density of ±40 C g^−1^)) and PPyDBS-PT8 (current densities ± 0.23 A g^−1^ to ±9.2 A g^−1^ (constant charge density of ±46 C g^−1^)) were conducted. The potential time curves of PPyDBS-PT4 in the three aqueous electrolytes (LiCl, NaCl, and KCl) at ±0.4 A g^−1^ are presented in Figure 5a. The specific capacitance calculated from Equation (1) against the current density i/m is shown in Figure 5b. The potential time curves of PPyDBS-PT8 (LiCl, NaCl, and KCl) at the applied current density ± 0.43 A g^−1^ is displayed in Figure 5c, and the specific capacitance against the current densities i/m is shown in Figure 5d. Long-term measurements (5000 cycles, ±8.0 A g^−1^) of PPyDBS-PT4 are presented at Figure S3a at the applied electrolytes LiCl, NaCl, and KCl to determine capacity retention. For PPyDBS-PT8, those are shown at Figure S3b (5000 cycles, ±9.2 A g^−1^).

The potential time curves of PPyDBS-PT4 (Figure 5a) reveal if the overlapping of the two subsequent cycles for each electrolyte is concurrent, showing that charging/discharging is in control [48]. The profile of each chronopotentiogram shows similar potentials for LiCl and NaCl, while the KCl potential time curves exhibit much higher potential. The reason for such differences can be found in the higher resistivity (lower conductivity) of the PPyDBS-PT4 films, as shown in Table 2 (nearly 1.8 times lower conductivity than LiCl). From Equation (1), by taking the slope at potential time curves discharging (after IR drop) at each current density i/m, the specific capacitance C_S_ was determined. The results are presented in Figure 5b with the best specific capacitance at ±0.20 A g^−1^ found for LiCl at 260.6 ± 21 F g^−1^, followed by NaCl with 231.1 ± 20 F g^−1^, and the lowest in this row was shown for KCl at 88.4 ± 7 F g^−1^. The relatively low specific capacitance of PPyDBS-PT4 in KCl is the reason for the higher potential at a constant charge of ±40 C g^−1^ (Figure 5a) with more deep slopes compared to LiCl and NaCl. 

In the case of PPyDBS-PT8 films, among the potential time curves (Figure 5c) of the three electrolytes, LiCl has the lowest potential, followed by NaCl and KCl with increasing potentials. When comparing the discharging curves after the IR drop, the NaCl revealed more flat lines (the slope will be smaller) in comparison to LiCl. The results of the specific capacitance calculations are shown in Figure 5d, with PPyDBS-PT8 measured in NaCl electrolyte having a specific capacitance of 154.7 ± 13 F g^−1^ (±0.23 A g^−1^), followed by LiCl with 111.2 ± 10 F g^−1^, and the lowest is shown for KCl with 75.3 ± 6.7 F g^−1^. The specific capacitance of PPyDBS-PT8 is much lower in all the applied electrolytes than those found for PPyDBS-PT4, shown from previous research [8] in similar PT content with the same tendency in aqueous electrolyte at ±0.09 A g^−1^ where 223 ± 20 F g^−1^ for PPyDBS-PT5 and 138.5 ± 13 F g^−1^ for PPyDBS-PT10 was obtained. Other research [32] using different POM materials showed that polyaniline PANI-PMo_12_ composites in aqueous electrolytes had specific capacitance at 170 F g^−1^ while combination with PEDOT reduced to 80 F g^−1^. Ternary nano-hybrids using PMo_12_ with reduced graphene oxide (rGO) and PPy showed 360 F g^−1^ at 0.5 A g^−1^ in 0.5 M sulfuric acid [49]. The combination of two POM additives (vanadium substituted PTA and molybdic acid) with PPy had specific capacitance at 294.8 F g^−1^ (1 A g^−1^, 0.25 M H_2_SO_4_) [50], similar to our results for PPyDBS-PT4 obtained from aqueous LiCl.

Additionally, the long-term measurements of 5000 cycles for PPyDBS-PT4 (8.0 A g^−1^) are presented in Figure S3a and for PPyDBS-PT8 (9.2 A g^−1^) in Figure S3b. PPyDBS-PT4 in aqueous LiCl (Figure S3a) at cycle 5 showed 122 ± 9 F g^−1^ which decreased to 108 ± 8.5 at cycle 5000, showing the best capacity retention of 88.5%. The capacity retention for NaCl of PPyDBS-PT4 was 82%, and for KCl, 57%. The PPyDBS-PT8 film, as shown for maximum specific capacitance in Figure 5d, was found best for NaCl with specific capacitance at cycle 5 (Figure S3b) was 63 ± 5.5 F g^−1^ (±9.2 A g^−1^), which was found at cycle 5000 at 47.6 ± 4 F g^−1^ having a capacity retention of 75.4%. The capacity retention for LiCl was 79%, and KCl was 45.7%. Other research [9] reported capacity retention using PW_12_@PPy and PMo_12_@PPy of 83–84% after 5000 cycles at 11 mA cm^−2^. The combination of POM with polymerizable ionic liquids (PILs) and rGO had a specific capacitance of 408 F g^−1^ (0.5 A g^−1^, 0.5 M H_2_SO_4_) and a specific capacity retention of 92% (10 A g^−1^) after 2000 cycles. In summary, POM combination with PPy, as shown in this research, has great potential in supercapacitor applications [51] with good capacity retention. 

### 3.5. Sensor Calibration

**Figure 6:**
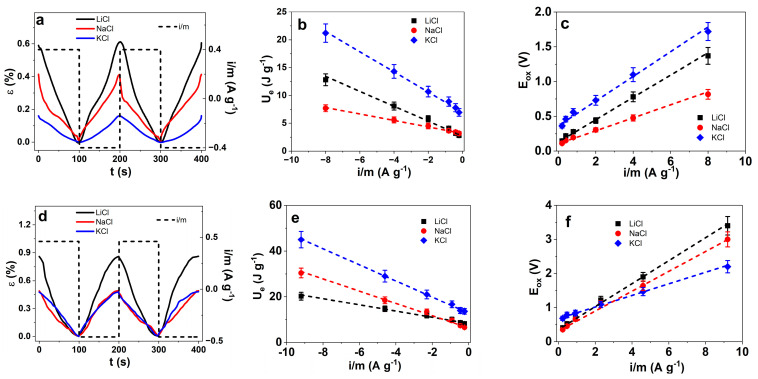
Chronopotentiometric measurements of the PPyDBS-PT composites using three different aqueous electrolytes such as LiCl (black line, ■), NaCl (red line, ●), and KCl (blue line, ◆). The strain time curves (two subsequent cycles, 3rd–4th) at the current density i/m of ±0.4 A g^−1^ (black dashed line) of PPyDBS-PT4 are presented in (**a**). From Equation (2), the electrical energy (U_e_) at discharging against current densities i/m is shown in (**b**) and the maximum potential at oxidation (E_ox_) against current densities is displayed in (**c**). The PPyDBS-PT8 potential time curves (3rd–4th cycles) at ±0.46 A g^−1^ (dashed black line) are shown in (**d**). The electrical energy U_e_ is presented in (**e**) and the potential at oxidation E_ox_ in (**f**) against the current densities i/m. The dashed lines in (**b**–**e**) represent the linear fits with the R^2^ correlation coefficient between 0.98 and 0.99.

PPy composites having the same charge densities at varied current densities as shown for PPyDBS-PT4 (±40 C g^−1^) and PPyDBS-PT8 (±46 C g^−1^) using the same electrical wires can detect different measurements beneath their linear actuation such as potential evolution and current. Only if the charging/discharging of those systems are in balance can those sensor functions be compared [52]. Here, we applied three different aqueous electrolytes: LiCl, NaCl, and KCl. In the above investigation, the order LiCl > NaCl > KCl was found for the highest solvation numbers in the PPy membranes [21], linear actuation properties, electronic surface conductivity, and specific capacitance (mainly followed by PPyDBS-PT4). Here, we want to explore whether we can differentiate the Li^+^, Na^+^, and K^+^ alkali ions. The strain against time curves of the chronopotentiometric measurements of the PPyDBS-PT4 films (LiCl, NaCl, and KCl) are shown in Figure 6a (current density i/m at ±4.0 A g^−1^). From the potential time curves (Figure 5a), the discharging curves are integrated at each applied current density i/m, and from Equation (2), the electrical energy U_e_ was calculated. The electrical energy U_e_ against the applied current density i/m is shown in Figure 6b. The maximum potential at oxidation E_ox_ from the potential time curves is plotted against the current densities (Figure 6c). The PPyDBS-PT8 films are presented in similar order with strain curves at the current density ±0.43 A g^−1^ of the three different applied electrolytes shown in Figure 6d, followed by electrical energy U_e_ (Figure 6e) and the potential evolution at oxidation E_ox_ (Figure 6f) against the current densities i/m. 

Comparing Figure 6a of the PPyDBS-PT4 strain time curves and Figure 6d of PPyDBS-PT8, there are differences in strain magnitude from the three different applied aqueous electrolytes. The PPyDBS-PT4 (Figure 6a) shows the separation of strain magnitude following the order LiCl (0.61%) > NaCl (0.42%) > KCl (0.16%). Having the same charge density (±40 C g^−1^), only the incorporated solvated cations (Li^+^, Na^+^, and K^+^) determine the expansion at the reduction of PPyDBS-PT4 films. Each electrolyte should give a similar strain from the ESCR model at the same charge density and varied current densities [18], as documented in Figure S4a. The strain time curves of PPyDBS-PT8 in Figure 6d are similar for NaCl (0.49%) and KCl (0.48%), while LiCl (0.86%) can be differentiated from the two other electrolytes, with strain against the current density at Figure S4b confirming the nearly constant strain for each electrolyte.

Previous research [53] investigated the phenomena of solvation, osmotic pressure [1], and ion speed inside PPyDBS of LiCl, NaCl, and CsCl regarding the tendency of Li^+^ > Na^+^ > Cs^+^ in linear actuation. In the PPyDBS films, the mobility of such alkali ions is different. The main explanation also considering our PPyDBS-PT composites is the interaction of affinity of ions to stored water in PPyDBS. For example, some larger ions such as Cs^+^ are not solvated inside PPyDBS, and the expected ion mobility should be high, but it was found that those large cations also, in our case K^+^ (solvated with 2.2 water molecules, Table 1), have very limited ion mobility. This leads to less opening of the polymer chains with PPyDBS as well as those that have a higher young modulus [1], as shown in Table 2 and Table 3 for Young’s modulus before and after actuation for PPyDBS-PT4 and PPyDBS-PT8 for aqueous KCl. Having metal alkali ions such as Li^+^, their affinity to hydration (Table 1) is the highest, followed by Na^+^, and it was discovered that inside PPyDBS at reduction, those ions are solvated up to 20 water molecules for Na^+^ and even higher for Li^+^ molecules which leads to higher expansion than K^+^ in PPyDBS-PT composites (Figure 3a, Figure 4a and Figure 6a,d). In this work, we also have another parameter that PPyDBS-PT8 is stiffer than PPyDBS-PT4, which shows in Figure 6d that Na^+^ and K^+^ have the same linear actuation at constant charge, leading to the assumption that the denser PPyDBS-PT8 influence the ion mobility of Na^+^ and K^+^.

The linear fit equations are presented in Table 4 to elaborate the sensor functions if the cations Li^+^, Na^+^, and K^+^ can be differentiated. PPyDBS-PT4 at a constant charge density of ±40 C g^−1^ and PPyDBS-PT8 at a constant charge density ± 46 C g^−1^ with the linear fit equations for either electrical energy (Figure 6b,e), potential at oxidation (Figure 6c,f), or strain (Figure S4a,b) are shown in Table 4.

From the linear fit equations in Table 4, some conclusions can be drawn regarding the differentiation of alkali cations found for PPyDBS-PT4, the tendency Li^+^ > Na^+^ > K^+^ in strain response (Figure S4a), and that they can be sensed from each other. Other linear equations, either the electrical energy U_e_ (Figure 6b) or the potential at oxidation E_ox_ (Figure 6c), have different linear fits and can be ascertained. When comparing the linear fits of PPyDBS-PT8, the strain of NaCl and KCl are nearly identical (Table 4) while the potential at oxidation E_ox_ (Figure 6f) has similar linear fits for LiCl and NaCl, and the electrical energy U_e_ (Figure 6e) shows some differentiations. 

Considering all the sensor equations, PPyDBS-PT4 shows electrical energy, potential at oxidation, and possible alkali cation differentiations in all the linear fits. Several properties of PPyDBS-PT4, such as best electronic conductivity, higher charge density due to more PT^3−^ incorporation, less dense films, and lower Young’s modulus after actuation are more supporting the alkali ions detection regarding Li^+^, Na^+^, and K^+^ in comparison to PPyDBS-PT8. The high Li^+^ and Na^+^ energy storage, as well as differentiations in sensor equations, give direction for possible applications for the separation of such alkali ions [54] or the use of those alkali ions in rechargeable batteries [55], capacitive deionization [56], and energy storage devices [57]. 

## 4. Conclusions

The composites PPyDBS-PT4 and PPyDBS-PT8 underwent electrochemical characterizations by applying aqueous electrolytes LiCl, NaCl, and KCl. For the PPyDBS-PT composites, the linear actuation revealed expansion at oxidation following the cation-driven actuation. During electropolymerization, the different PTA concentrations (4 mM and 8 mM) revealed that the higher concentration of PTA did not lead to a higher amount of PT^3−^ in PPyDBS, while some PTA are used to catalyze the PPyDBS-PT8 composite, forming denser films (7.4% more dense than PPyDBS-PT4). Those differences in PT^3−^ concentration in PPyDBS (PPyDBS-PT4) showed 3.5–3.8 times higher charge density, 3–4 times lower Young’s modulus before actuation, and 3 times higher electronic conductivity that affects the linear actuation properties of 1.3–2.5 times higher strain of LiCl > NaCl > KCl. The specific capacitance of PPyDBS-PT4 had the best result for LiCl with 260.6 ± 21 F g^−1^ (±0.2 A g^−1^) capacity retention (5000 cycles at ±8.0 A g^−1^) of 88.5%. The PPyDBS-PT8 specific capacitance was 2.3 times (111.2 ± 10 F g^−1^ at 0.23 A g^−1^) lower using the same electrolyte LiCl and a capacity retention of 79%. Further investigation was conducted by applying the chronopotentiometry measurements of PPyDBS-PT4 having a constant charge density of ±40 C g^−1^ and PPyDBS-PT8 with ±46 C g^−1^ with the main goal to find out if the metal alkali cation Li^+^, Na^+^, and K^+^ can be differentiated from each other. Considering the size of the cations (Li^+^, Na^+^, and K^+^) as well their solvation shells but also the different compactness of the composites, the PPyDBS-PT4 in comparison to PPyDBS-PT8 revealed that all three metal alkali ions can be sensed from each other using sensor equation with either the electrical energy, the potential at oxidation, or the strain response at varied current densities. The multifunctional PPyDBS-PT4 composite as the actuator, sensor, and energy storage device can be applied as actuators in soft robotics, electrode materials for batteries, capacitive deionization membranes, and supercapacitors.

## Figures and Tables

**Table 1:** Aqueous electrolytes regarding non-hydrated radius r, apparent dynamic hydration numbers N, coordination number in H_2_O (N_H2O_), and coordination number of ions in membrane PPy (N_PPy_).

Electrolytes Cation	r (Å) [40]	N [40]	$N_{H_{2}O}$ [41]	N_PPy_ [21]
Li+	0.76	0.58	4	5.3–5.5
Na+	1.02	0.22	5	4.3–4.5
K+	1.38	0	6	2.0–2.2

**Table 2:** Strain ε, charge densities Q, electronic conductivity σ_e_, and elastic modulus Y before and after actuation of PPyDBS-PT4 in aqueous electrolytes LiCl, NaCl, and KCl.

PPyDBS-PT4 Applied Aqueous Electrolytes	ε (%)	Q (C cm^−3^)	σ_e_ (S cm^−1^)	Y (MPa)	
				Before	After
LiCl	6.6 ± 0.4	149.2 ± 12.2	13.5 ± 0.9	4.8 ± 0.3	1.4 ± 0.1
NaCl	4.7 ± 0.3	137.6 ± 11.1	10.3 ± 0.6	4.4 ± 0.4	1.1 ± 0.1
KCl	2.4 ± 0.2	114.5 ± 9.4	7.4 ± 0.4	5.0 ± 0.4	4.2 ± 0.3

**Table 3:** PPyDBS-PT8 films at the three aqueous electrolytes: comparison of strain ε, charge density Q, electronic conductivity σ_e_, and elastic modulus Y (before and after actuation).

PPyDBS-PT8 Applied Aqueous Electrolytes	ε (%)	Q (C cm^−3^)	σ_e_ (S cm^−1^)	Y (MPa)	
				Before	After
LiCl	2.6 ± 0.2	41.2 ± 2.8	7.2 ± 0.5	18.5 ± 1.3	11.3 ± 0.8
NaCl	2.3 ± 0.1	36.4 ± 2.2	6.2 ± 0.4	17.2 ± 1.2	11.5 ± 0.7
KCl	1.8 ± 0.1	32.6 ± 2.1	3.8 ± 0.3	20.4 ± 1.4	18.5 ± 1.4

**Table 4:** Linear fit equations of PPyDBS-PT4 and PPyDBS-PT8 of the applied electrolytes showing electrical energy U_e,_ potential at oxidation E_ox_ and strain ε.

Electrolytes	U_e_ (J g^−1^)	E_ox_ (V)	ε (%)
* PPyDBS-PT4			
LiCl	$2.7-1.34\frac{i}{m}\;(Ag^{-1})$	$0.13+0.16\frac{i}{m}\;(Ag^{-1})$	0.60 ± 0.050
NaCl	$3.2-0.60\frac{i}{m}\;(Ag^{-1})$	$0.11+0.09\frac{i}{m}\;(Ag^{-1})$	0.41 ± 0.037
KCl	$7.0-1.8\frac{i}{m}\;(Ag^{-1})$	$0.36+0.18\frac{i}{m}\;(Ag^{-1})$	0.16 ± 0.015
** PPyDBS-PT8			
LiCl	$8.3-1.35\frac{i}{m}\;(Ag^{-1})$	$0.36+0.34\frac{i}{m}\;(Ag^{-1})$	0.85 ± 0.079
NaCl	$6.3-2.69\frac{i}{m}\;(Ag^{-1})$	$0.32+0.29\frac{i}{m}\;(Ag^{-1})$	0.52 ± 0.048
KCl	$12.8-3.53\frac{i}{m}\;(Ag^{-1})$	$0.68+0.17\frac{i}{m}\;(Ag^{-1})$	0.44 ± 0.04

* PPyDBS-PT4: current density i/m: ±0.2 A g^−1^, ±0.4 A g^−1^, ±0.8 A g^−1^, ±2.0 A g^−1^, ±4.0 A g^−1^, and ±8.0 A g^−1^. Charge density: ±40 C g^−1^. ** PPyDBS-PT8: current density i/m: ±0.23 A g^−1^, ±0.46 A g^−1^, ±0.92 A g^−1^, ±2.3 A g^−1^, ±4.6 A g^−1^, and ±9.2 A g^−1^. Charge density: ±46 C g^−1^.

## Data Availability

The original contributions presented in this study are included in the article/Supplementary Material. Further inquiries can be directed to the corresponding author.

## Supplementary Materials

The following supporting information can be downloaded at https://www.mdpi.com/article/10.3390/polym17030262/s1, Figure S1. EDX spectrum of PPyDBS-PT4 after cyclic voltammetric linear actuation measurements at oxidation (0.65 V, black line) and reduction (−0.6 V, red line) showing in (a) LiCl, (b) NaCl, and (c) KCl; Figure S2. EDX spectrum of PPyDBS-PT8 after cyclic voltammetric linear actuation measurements at oxidation (0.65 V, black line) and reduction (−0.6 V, red line) showing in (a) LiCl, (b) NaCl, and (c) KCl; Figure S3. Chronopotentiometric long-term measurements (5000 cycles, 0.1 Hz) in aqueous LiCl (■), NaCl (●), and KCl (◆) showing in (a) PPyDBS-PT4 at ±8.0 A g^−1^ and in (b) PPyDBS-PT8 (±9.2 A g^−1^); Figure S4. Chronopotentiometric measurements of strain ε in aqueous electrolytes LiCl (■), NaCl (●), and KCl (◆) against applied current density i/m showing in (a) PPyDBS-PT4 and in (b) PPyDBS-PT8.
