# Supplementary material for: Alkali Metal Ion Insertion in Polypyrrole Polyoxometalates for Multifunctional Actuator–Sensor–Energy Storage Devices
Source: Polymers (Basel). 2025 Jan 21;17(3):262. doi: 10.3390/polym17030262 (PMC11820236; doi:10.3390/polym17030262)
Supplement: Supplementary file 1 [file polymers-17-00262-s001.zip › polymers-3415768-supplementary.docx]

**Supplementary**

**Alkali metal ions insertion in polypyrrole polyoxometalates for multifunctional actuator-sensor-energy storage devices**

Rudolf Kiefer^1,*^, Ngoc Tuan Nguyen^2^, and Quoc Bao Le^1^

^1^Conducting polymers in composites and applications Research Group, Faculty of Applied Sciences, Ton Duc Thang University, Ho Chi Minh City, Vietnam

^2^Faculty of Applied Sciences, Ton Duc Thang University, Ho Chi Minh City, Vietnam

*Corresponding author. Tel: +84 792696724. E-mail: rudolf.kiefer@tdtu.edu.vn (Rudolf Kiefer)


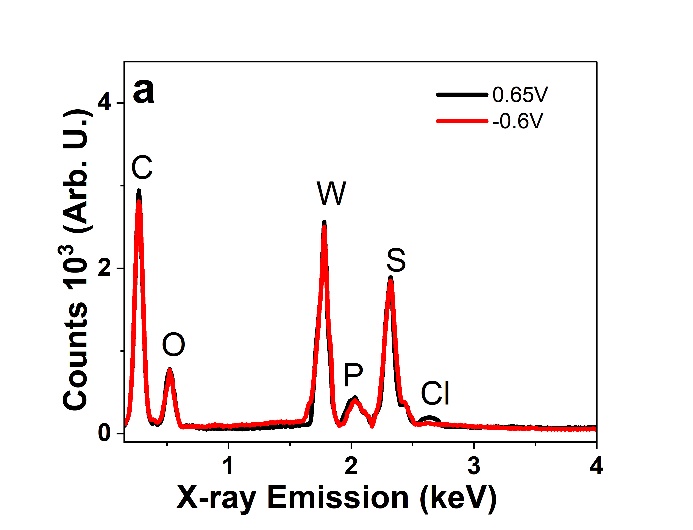

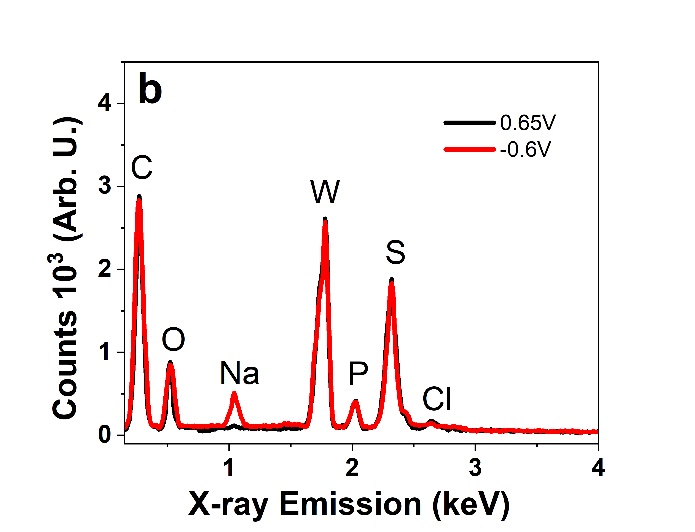

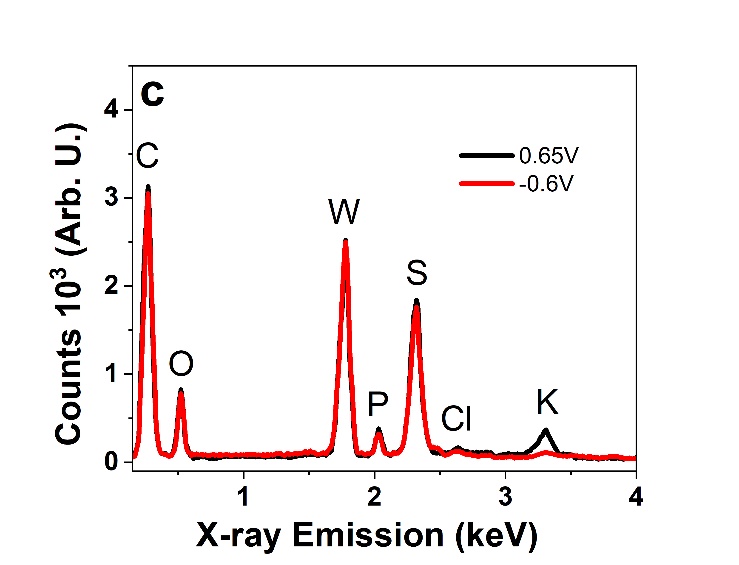


Figure S1. EDX spectrum of PPyDBS-PT4 after cyclic voltammetric linear actuation measurements at oxidation (0.65 V, black line) and reduction (-0.6 V, red line) showing in a) LiCl, b) NaCl and c) KCl.


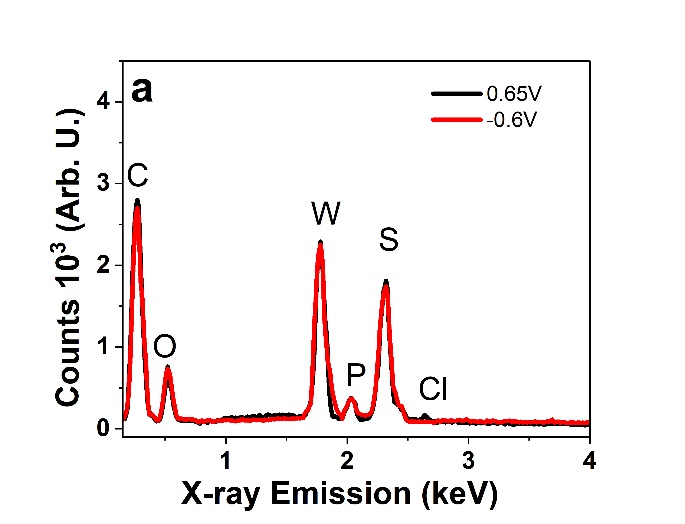

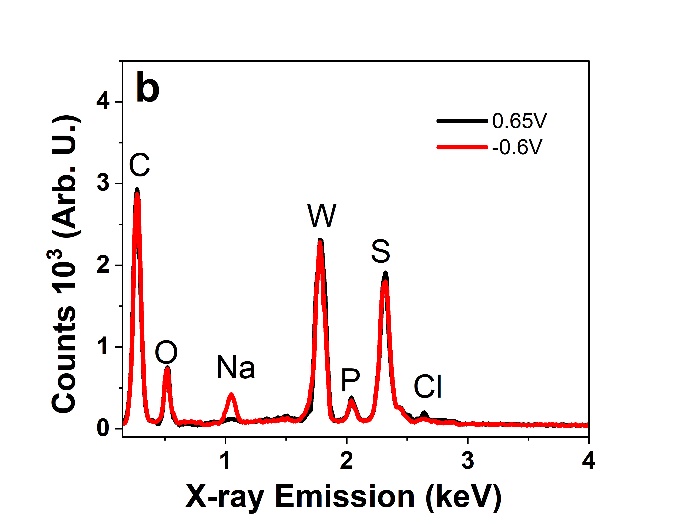

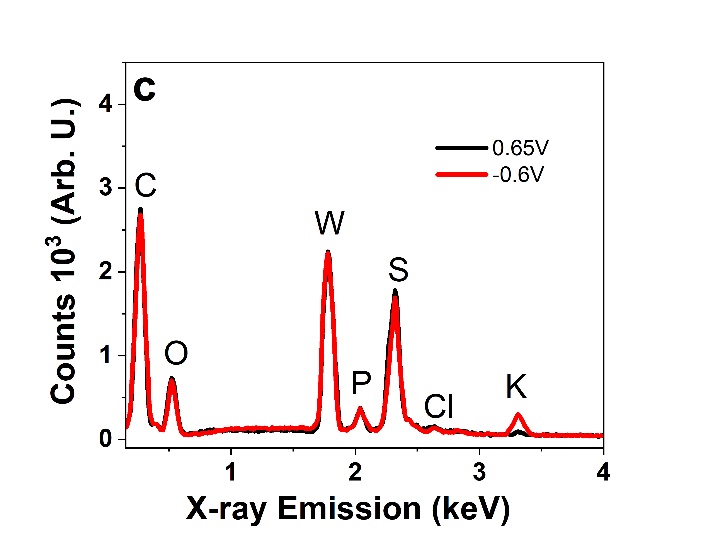


Figure S2. EDX spectrum of PPyDBS-PT8 after cyclic voltammetric linear actuation measurements at oxidation (0.65 V, black line) and reduction (-0.6 V, red line) showing in a) LiCl, b) NaCl and c) KCl.


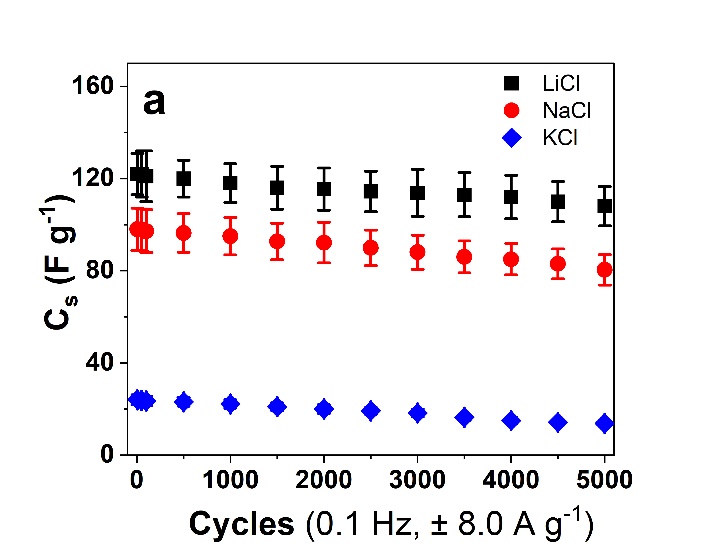

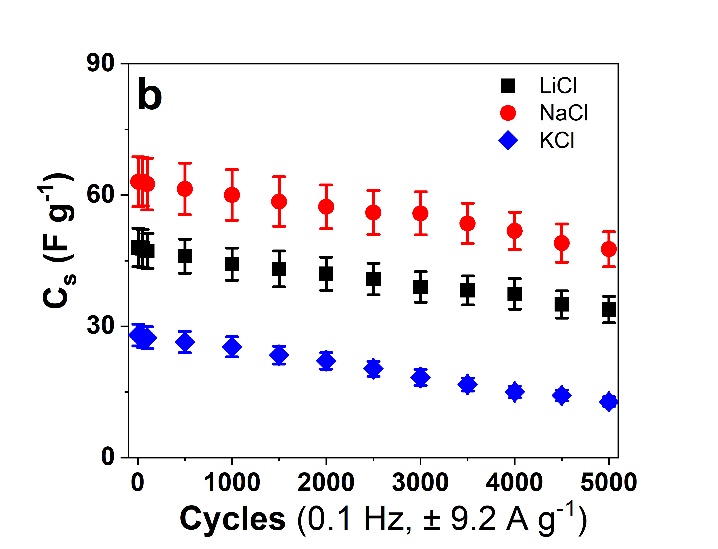


Figure S3. Chronopotentiometric long-term measurements (5000 cycles, 0.1 Hz) in aqueous LiCl (■), NaCl (●), and KCl (◆) showing in a) PPyDBS-PT4 at ± 8.0 A g^-1^ and in b) PPyDBS-PT8 (± 9.2 A g^-1^).


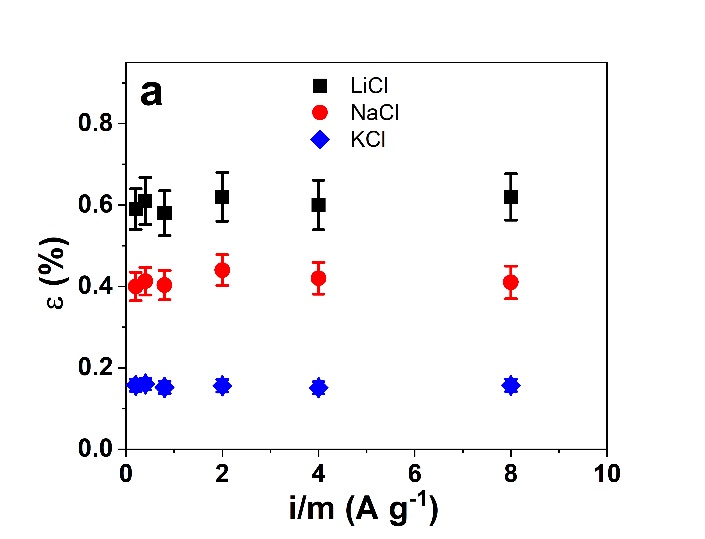

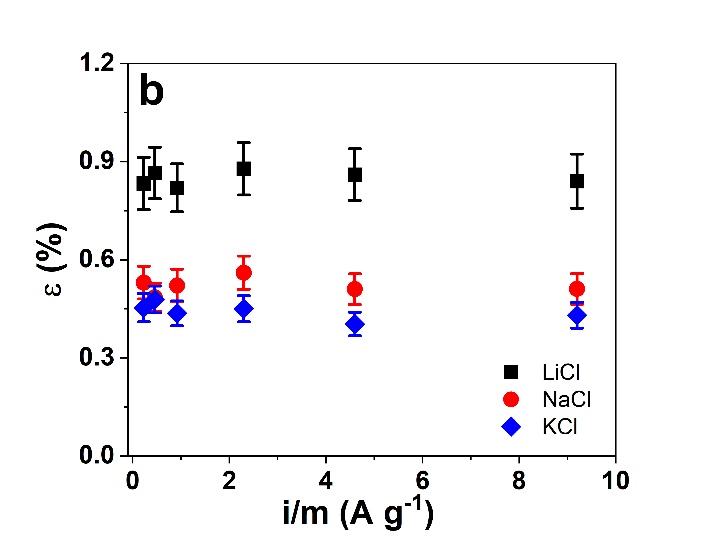


Figure S4. Chronopotentiometric measurements of strain ε in aqueous electrolytes LiCl (■), NaCl (●) and KCl (◆) against applied current density i/m showing in a) PPyDBS-PT4 and in b) PPyDBS-PT8.
